# Association of neonatal hypothermia with neonatal hypoglycemia

**DOI:** 10.3389/fendo.2025.1641140

**Published:** 2025-08-27

**Authors:** Henrike Hoermann, Marcia Roeper, Lisa Friesl, Calvin Kurz, Juliane Tautz, Mark Dzietko, Ertan Mayatepek, Thomas Meissner, Sebastian Kummer

**Affiliations:** Department of General Pediatrics, Neonatology and Pediatric Cardiology, Medical Faculty, University Hospital Düsseldorf, Heinrich-Heine-University, Düsseldorf, Germany

**Keywords:** hypoglycemia, hypothermia, neonate, newborn, glucose

## Abstract

**Introduction:**

About 15% of neonates suffer from hypoglycemia. Hypothermia is associated with hypoglycemia; however, there are limited empiric data analyzing this association. Accordingly, hypothermia is not listed as a risk factor in many hypoglycemia guidelines. This study aimed to analyze hypothermia in regard to neonatal hypoglycemia.

**Methods:**

Prospective study of 1018 neonates ≥35 + 0 weeks. Neonates at-risk for hypoglycemia (n=857) received a standardized blood glucose (BG) screening/management. Controls (n=161) received at least two BG measurements at 2–3 and 36–72 hours. Rectal temperature was measured at 1–3 hours, upon transfer to the maternity/pediatric ward, and at clinical discretion (hypothermia = <36.5°C).

**Results:**

236/1018 (23.2%) neonates had at least one episode of hypothermia. More hypothermic compared to non-hypothermic neonates had hypoglycemia ≤2.5 mmol/l (≤45 mg/dl) (53.4% vs. 26.2%, P<.001) and <1.7 mmol/l (<30 mg/dl) (12.7% vs. 1.4%, P<.001), and subsequently required treatment more frequently. Small for gestational age (SGA) and/or fetal growth restriction (FGR), prematurity and perinatal stress were associated with a higher risk for hypothermia. In SGA and/or FGR neonates the incidence of hypoglycemia ≤2.5 mmol/l (≤45 mg/dl) and <1.7 mmol/l (<30 mg/dl) was higher for hypothermic compared to non-hypothermic neonates (58% vs. 35%, P<.001 and 15% vs. 4%, P=.003).

**Conclusion:**

Hypothermia was strongly associated with neonatal hypoglycemia, leading to more frequent hypoglycemic episodes and a greater need for treatment. Further prospective studies are needed to elucidate the direction of causality between both conditions and to assess the effectiveness of thermal management strategies in reducing hypoglycemia. Awareness should be raised to rule out hypoglycemia in case of hypothermia, and vice versa.

## Introduction

Neonatal hypothermia is a common problem that is associated with higher mortality and morbidity in newborns and is one of the major therapeutic challenges in the postnatal period (1, 2). Neonatal hypothermia is defined by the World Health Organization as a body temperature <36.5°C (3). In very low birth weight neonates (<1500 g) a 1°C drop of the admission temperature to the neonatal intensive care unit was associated with a 28% increased risk of mortality (4). Whereas most studies focus on thermoregulation in preterm infants, data on late preterm or term born neonates are rarer, especially regarding data on neonatal hypothermia in high-income countries (5). Reported incidence of neonatal hypothermia in late preterm and term born neonates varies from 21.7% to 83% with higher rates in developing countries (2, 5, 6).

Hypothermia is associated with neonatal hypoglycemia, even though the direction of causality is not fully elucidated. With 15% of all newborns developing postnatal hypoglycemia, it is the most common metabolic problem in neonates which can lead to brain damage and suboptimal neurodevelopment (7, 8). Several studies analyzing hypoglycemia risk factors found that hypothermia was associated with an increased risk of developing neonatal hypoglycemia (9–14). However, most of these studies focused on the identification of hypoglycemia risk factors and did not analyze the relationship between hypothermia and hypoglycemia in detail. Moreover, some papers on neonatal hypothermia did not even analyze or report a possible association between hypothermia with hypoglycemia (5). Furthermore, hypothermia is only mentioned as a hypoglycemia risk factor in 8/13 national hypoglycemia guidelines, indicating insufficient awareness for this association (15).

The aim of this study was to analyze hypothermia in late preterm and term born neonates with respect to neonatal hypoglycemia.

## Methods

### Study design and participants

We conducted a prospective monocentric analysis in 1018 neonates at a tertiary university hospital. The study consists of 3 interrelated studies (16–18), in which all neonates where uniformly treated regarding blood glucose (BG) screening and thermal management throughout the recruitment period.

Neonates were enrolled from 05/2020 to 09/2022 at the University Hospital Düsseldorf. Neonates with one or more of the following risk factors were assigned to the risk group: small or large for gestational age (SGA; LGA) (birth weight <10^th^/>90^th^ centile); fetal growth restriction (FGR) [Gordijn et al. (19)]; maternal/gestational diabetes; prematurity (35 + 0-36 + 6 weeks of gestation); maternal risk factors (including preeclampsia, hypertension, medication with beta-blockers, antidepressants or metformin); perinatal stress (asphyxia, respiratory distress, arterial umbilical cord blood pH <7.1, vacuum extraction/unplanned cesarean due to pathological cardiotocography); hypothermia. Neonates without a risk factor were assigned to the control group. Neonates at-risk for hypoglycemia received a standardized BG screening and management according to local standard operating procedure (SOP) (18). The control group received at least two BG measurements at the age of 2–3 hours and at the age of 36–72 hours. In case of low BG levels further measures were taken according to the hypoglycemia SOP (18). As part of the clinical routine rectal body temperature was measured in the delivery room (age: 1–3 hours), after transferal to the maternity or pediatric ward, and at clinical discretion. Hypothermia was defined as a rectal temperature <36.5° (3); mild hypothermia: 36.0-36.4°C; moderate/severe hypothermia: <36.0°C. Neonates without a documented temperature measurement (n=7) were assigned to the non-hypothermic group as it was assumed that there were no clinical signs of hypothermia that would have warranted a temperature measurement. Ambient outside temperature values for Düsseldorf were derived from *www.wetterkontor.de* *; Deutscher Wetterdienst* (Access date: March 26, 2025). If recruitment took place in the same month in two or three years, the mean temperature was used for analysis. The maternity ward had no air conditioning.

Clinical data were obtained from the medical files. Data were recorded with Claris FileMaker Pro version 19 (Claris International Inc).

The studies were approved by the ethics committee of the Medical Faculty of the Heinrich Heine University Düsseldorf according to the Declaration of Helsinki. Both parents of each neonate gave written informed consent.

### Statistical analysis

Statistical analyses were performed using IBM SPSS Statistics version 29.0.2.0 (20) (IBM Corp). χ^2^ and Fisher exact tests were used to analyze categorical variables. Medians were compared using Mann-Whitney U test with Bonferroni correction. Medians with IQRs are reported.

A mixed linear model was conducted to estimate whether body temperatures correlate with the BG levels, using subjects and age (minutes) as random effects. Binary logistic regression analyses were used to predict hypothermia onset, based on different hypoglycemia risk factors. Spearman correlation was computed to test for an association between the ambient temperature in the birth month and the percentage of neonates with hypothermia in the respected month.

## Results

A total number of 1018 neonates were included (46.2% female; 161 controls, 857 at-risk).

The majority (1011/1018 (99.3%)) received at least one documented temperature measurement and 236/1018 (23.2%) had at least one hypothermic episode. Median age at first episode of hypothermia (documented in 215/236 neonates) was 321 (161, 709) minutes (**Table 1**). Of 236 neonates, 190 neonates had mild hypothermia and 46 neonates moderate/severe hypothermia. Clinical characteristics did not differ between the group of mild and moderate/severe hypothermia (**Supplementary eTable 1**).

**Table 1:** Summary of temperature measurements and hypothermic episodes of all neonates.

	All neonates (n=1018)number/total number (%)
At least one temperature measurement	1011/1018 (99.3%)
Temperature measurement in the delivery room	910/1018 (89.4%)
Body temperature in the delivery room (°C) [median (IQR)]	37.1 (36.9; 37.3)
At least one episode of hypothermia	236/1018 (23.2%)
Hypothermia in the delivery room	23/236 (9.8%)
Number of temperature measurements at the maternity ward [median (IQR)]	3 (2, 5)
Body temperature after transfer to the maternity ward [median (IQR)]	37.0 (36.8; 37.2) (n=905)
Neonates who received thermal therapy (warming bed/incubator) at the maternity ward	261/1018 (25.6%)
Body temperature after transfer to the children’s hospital (°C) [median (IQR)]	37.0 (36.5; 37.3) (n=170)
Age at first onset of hypothermia (if documented) (minutes) [median (IQR)]	321 (161; 709) (n=215)

IQR, interquartile range.

At the maternity ward, a total of 261 neonates were temporarily placed in a warming bed/incubator. Clinical characteristics for neonates with and without hypothermia are shown in **Table 2**.

**Table 2:** Clinical characteristics of neonates with and without hypothermia.

	No hypothermia n=782number/total number (%)	Hypothermia n=236number/total number (%)	P
Weeks of gestation [median (IQR)]	39 + 0 (38 + 0; 40 + 1)	38 + 4 (37 + 0; 39 + 5)	.001^a,c^
Female	372/782 (47.6%)	98/236 (41.5%)	.10^b^
Delivery mode			
Vaginal	294/782 (37.6%)	117/236 (49.6%)	.001^b,d^
Elective cesarean	291/782 (37.2%)	64/236 (27.1%)	.004^b^
Unplanned cesarean	197/782 (25.2%)	55/236 (23.3%)	.56^b^
Birth weight (g) [median (IQR)]	3380 (2899; 3870)	2863 (2580; 3338)	<.001^a,c^
Birth weight SDS [median (IQR)]	-0.04 (-0.83; 0.98)	-0.78 (-1.56; 0.03)	<.001^a,c^
APGAR 1; 5; 10 minutes [median (IQR)]	9 (9; 9), 10 (10; 10), 10 (10; 10)	9 (9);, 10 (9; 10), 10 (10; 10)	.12;.05;.15^a^
Arterial cord blood pH [median (IQR)]	7.28 (7.24; 7.32)	7.28 (7.22; 7.32)	.58^a^
Arterial cord blood base excess (mmol/l) [median (IQR)]	-3.3 (-6.4; -1.7)	-4.1 (-6.65; -2.0)	.08^a^
Feeding type			
Breastmilk	101/782 (12.9%)	7/236 (3.0%)	<.001^b,d^
Breastmilk + formula	608/782 (77.8%)	201/236 (85.2%)	.01^b^
Formula	73/782 (9.3%)	28/236 (11.9%)	.26^b^
Transient need for nasogastric tube	37/782 (4.7%)	27/236 (11.4%)	.001^b,d^
Respiratory distress syndrome	43/782 (5.5%)	23/236 (9.8%)	.02^b^
Infection	22/782 (2.8%)	11/236 (4.7%)	.21^b^
Total number of hypoglycemia risk factors (hypothermia excluded) [median (IQR)]	1 (1; 2)	1(1; 2)	<.001^a,c^
No other risk factor	161/782 (20.6%)	28/236 (11.9%)	.003^b^
SGA and/or FGR	144/782 (18.4%)	95/236 (40.3%)	<.001^b,d^
LGA	142/782 (18.2%)	12/236 (5.1%)	<.001^b,d^
Prematurity	116/782 (14.8%)	53/236 (22.5%)	.007^b^
Maternal diabetes/gestational diabetes	291/782 (37.2%)	74/236 (31.4%)	.10^b^
Perinatal stress	134/782 (17.1%)	60/236 (25.4%)	.004^b^
Maternal risk factors	65/782 (8.3%)	19/236 (8.1%)	.90^b^
At least one episode of hypoglycemia ≤2.5 mmol/l (≤45 mg/dl)	205/782 (26.2%)	126/236 (53.4%)	<.001^b,d^
At least one episode of hypoglycemia <1.7 mmol/l (<30 mg/dl)	11/782 (1.4%)	30/236 (12.7%)	<.001^b,d^
Lowest measured blood glucose (mmol/l) [median (IQR)] [mg/dl]	2.9 (2.5; 3.3) [53 (45; 59)]	2.5 (2.0; 2.9) [45 (36; 53)]	<.001^a,c^
Number of BG levels between 2.6-3.0 mmol/l (46–54 mg/dl) [median (IQR)]	1 (0; 1)	1 (0; 2)	<.001^a,c^
Number of BG levels ≤2.5 mmol/l (≤45 mg/dl) [median (IQR)]	0 (0; 1)	1 (0; 1)	<.001^a,c^
Treatment at the children’s hospital	94/782 (12.0%)	76/236 (32.2%)	<.001^b,d^
Duration of hospital stay (days) [median (IQR)]	5 (3; 10)	7 (4; 11)	.11^a^
Transfer to the children’s hospital due to hypoglycemia	32/782 (4.1%)	41/236 (17.4%)	<.001^b,d^
Duration of treatment with intravenous glucose due to hypoglycemia (days) [median (IQR)]	2 (2; 3)	3 (2; 5)	.014^a^
Maximum intravenous glucose (mg/kg/min) [median (IQR)]	4.5 (3.4; 5.2)	4.7 (3.6; 5.4)	.18^a^
Treatment with glucose gel	214/782 (27.4%)	122/236 (51.7%)	<.001^b,d^

FGR, fetal growth restriction; IQR, interquartile range; LGA, large for gestational age; SDS, standard deviation score; SGA, small for gestational age.

^a^Mann-Whitney U test, ^b^χ2 or Fisher’s Exact test; both with Bonferroni correction; ^c^statistically significant at P<.0033; ^d^statistically significant at P<.0023.

### Risk factors for hypothermia

In the hypothermia group there was a significant higher percentage of SGA and/or FGR neonates compared to the non-hypothermic neonates (40.3% vs. 18.4%, P<.001) (**Table 2**). The comparison of the percentage of premature neonates and neonates with perinatal stress in the hypothermic vs. non-hypothermic neonates did not reach statistical significance after Bonferroni correction (22.5% vs. 14.8%, P=.007; 25.4% vs. 17.1%, P=.004) (**Table 2**). The percentage of LGA neonates was lower in the hypothermic compared to the non-hypothermic neonates (5.1% vs. 18.2%, P<.001) (**Table 2**). In line with these results, the multiple binary logistic regression analysis showed that the risk factors SGA and/or FGR, prematurity and perinatal stress were positively associated with an increased risk to develop hypothermia, whereas LGA was associated with a decreased risk to develop hypothermia (**Table 3**).

**Table 3:** Evaluation of the association between different hypoglycemia risk factors and the risk to develop hypothermia.

Risk factor	Odds ratio	95% CI	P^a^
SGA and/or FGR	2.59	1.84; 3.62	<.001
LGA	0.36	0.19; 0.67	.001
Prematurity	1.54	1.04; 2.26	.03
Maternal diabetes/gestational diabetes	0.99	0.71; 1.39	.97
Perinatal stress	1.45	1.01; 2.08	.045
Maternal risk factors	0.83	0.48; 1.46	.52

CI, confidence interval; FGR, fetal growth restriction; LGA, large for gestational age; SGA, small for gestational age.

^a^Multiple binary logistic regression analysis.

In the hypothermic group more neonates were born vaginally compared to the non-hypothermic group (49.6% vs. 37.6%, P=.001) (**Table 2**).

### Hypothermia and hypoglycemia

A total of 1350 BG/temperature pairs were analyzed. BG levels correlated positively with the body temperature (F(1, 1332.999)=99.191, P<.001). 202/566 (35.7%) BG values ≤2.5 mmol/l (≤45 mg/dl) and 29/54 (54%) BG values <1.7 mmol/l (<30 mg/dl) had a concomitant temperature documented.

The number of infants who developed at least one hypoglycemic episode ≤2.5 mmol/l (≤45 mg/dl) and <1.7 mmol/l (<30 mg/dl) was significantly higher in the hypothermic group compared to the non-hypothermic group (53.4% vs. 26.2%, P<.001 and 12.7% vs. 1.4%, P<.001) (**Table 2**). Moreover, the total number of BG levels between 2.6-3.0 mmol/l (46–54 mg/dl) and ≤2.5 mmol/l (≤45 mg/dl) was higher in hypothermic compared to non-hypothermic neonates (**Table 2**). Correspondingly, more neonates in the hypothermic group had to be transferred to the children’s hospital for hypoglycemia treatment compared to non-hypothermic neonates (17.4% vs. 4.1%, P<.001). Additionally, more neonates in the hypothermic group compared to the non-hypothermic group were treated with glucose gel (51.7% vs. 27.4%, P<.001) (**Table 2**). Fewer infants in the hypothermic group received exclusively breastmilk compared to the non-hypothermic group (3.0% vs. 12.9%, P<.001), and more neonates in the hypothermic group required transient feeding via a nasogastric tube (11.4% vs. 4.7%, P=.001) (**Table 2**).

The percentage of neonates that had at least one hypoglycemic episode ≤2.5 mmol/l (≤45 mg/dl) or <1.7 mmol/l (<30 mg/dl) and the lowest measured BG level did not differ significantly between mild and moderate/severe hypothermia (**Supplementary eTable1**).

### Subgroup analysis of controls and neonates with hypothermia as the only risk factor

In a subgroup analysis of control neonates without hypothermia and neonates with hypothermia as the only hypoglycemia risk factor, the minimal measured BG level was significantly lower in the hypothermic neonates compared to the control neonates without any hypoglycemia risk factor (2.6 (2.2; 2.9) vs. 3.1 (2.6; 3.6) mmol/l (46 (40; 52) vs. 56 (47; 64) mg/dl) P<.001) (**Supplementary eTable 2**). Furthermore, the percentage of neonates who had to be transferred to the children’s hospital was higher in the hypothermic vs. the non-hypothermic control neonates (18% vs. 2%, P=.002) (**Supplementary eTable 2**). None of the hypothermic neonates (0%) compared to the non-hypothermic control group (23%) received breastmilk only (P=.002). Correspondingly, the rate of neonates who received breastmilk plus formula was significantly higher in the hypothermic compared to the non-hypothermic control group (96% vs. 67%, P<.001) (**Supplementary eTable 2**). Treatment with glucose gel and transferal rate to the children’s hospital due to hypoglycemia were higher in the hypothermic group compared to the non-hypothermic control group but failed to reach statistical significance after Bonferroni correction (**Supplementary eTable 2**). Other clinical characteristics did not differ between both groups (**Supplementary eTable 2**).

### Subgroup analysis of SGA and/or FGR neonates

SGA and/or FGR neonates with and without hypothermia were compared. Clinical characteristics and the number of other risk factors did not differ between both the two groups (**Table 4**). However, the number of infants who developed at least one BG level ≤2.5 mmol/l (≤45 mg/dl) and <1.7 mmol/l (<30 mg/dl) was significantly higher in the hypothermic SGA and/or FGR neonates compared to the non-hypothermic SGA and/or FGR neonates (58% vs. 35%, P<.001 and 15% vs. 4%, P=.003) (**Table 4**). Moreover, the minimal ever measured BG level was lower in the hypothermic compared to the non-hypothermic SGA and/or FGR neonates (2.4 (2.0; 3.0) vs. 2.7 (2.3; 3.1) mmol/l (44 (36; 54) vs. 48 (41; 56) mg/dl), P=.002) (**Table 4**).

**Table 4:** Clinical characteristics of SGA and/or FGR neonates with and without hypothermia.

	SGA and/or FGR without hypothermia n=144number/total number (%)	SGA and/or FGR with hypothermia n=95number/total number (%)	P
Weeks of gestation [median (IQR)]	39 + 0 (37 + 6; 40 + 1)	38 + 6 (37 + 6; 40 + 1)	.75^a^
Female	63/144 (44%)	42/95 (44%)	.94^b^
Delivery mode			
Vaginal	55/144 (38%)	50/95 (53%)	.03^b^
Elective cesarean	51/144 (35%)	23/95 (24%)	.07^b^
Unplanned cesarean	38/144 (26%)	22/95 (23%)	.57^b^
Birth weight (g) [median (IQR)]	2673 (2456; 2905)	2650 (2320; 2850)	.34^a^
Birth weight SDS [median (IQR)]	-1.56 (-1.87; -1.37)	-1.66 (-2.02; -1.44)	.04^a^
APGAR 1; 5; 10 minutes [median (IQR)]	9 (9; 9); 10 (10; 10); 10 (10; 10)	9 (9; 9); 10 (10; 10); 10 (10; 10)	.93;.86;.93^a^
Arterial cord blood pH [median (IQR)]	7.28 (7.22; 7.32)	7.28 (7.22; 7.32)	.76^a^
Arterial cord blood base excess (mmol/l) [median (IQR)]	-3.6 (-6.8; -2.0)	-4.5 (-6.9; -2.55)	.32^a^
Feeding type			
Breast milk	12/144 (8%)	3/95 (3%)	.17^b^
Breast milk + formula	116/144 (81%)	80/95 (84%)	.50^b^
Formula	16/144 (11%)	12/95 (13%)	.84^b^
Transient need for nasogastric tube	19/144 (13%)	12/95 (13%)	.99^b^
Respiratory distress syndrome	4/144 (3%)	4/95 (4%)	.72^b^
Infection	4/144 (3%)	1/95 (1%)	.65^b^
Total number of hypoglycemia risk factors [median (IQR)] (hypothermia excluded)	2 (2; 3)	2 (1; 3)	.61^a^
Prematurity	26/144 (18%)	11/95 (12%)	.20^b^
Maternal diabetes/gestational diabetes	29/144 (20%)	16/95 (17%)	.61^b^
Perinatal stress	36/144 (25%)	20/95 (21%)	.48^b^
Maternal risk factors	13/144 (9%)	11/95 (12%)	.52^b^
At least one episode of hypoglycemia ≤2.5 mmol/l (≤45 mg/dl)	50/144 (35%)	55/95 (58%)	<.001^b,d^
At least one episode of hypoglycemia <1.7 mmol/l (<30 mg/dl)	5/144 (4%)	14/95 (15%)	.00258^b,d^
Number of BG levels between 2.6-3.0 mmol/l (46–54 mg/dl) [median (IQR)]	1 (0; 2)	1 (0; 2)	.54^a^
Number of BG levels ≤2.5 mmol/l (≤45 mg/dl) [median (IQR)]	0 (0; 1)	1 (0; 2)	<.001^a,c^
Lowest measured blood glucose (mmol/l) [median (IQR)] [mg/dl]	2.7 (2.3; 3.1) [48 (41; 56)]	2.44 (2.0; 3.0) [44 (36; 54)]	.002^a,c^
Treatment at the children’s hospital	36/144 (25%)	29/95 (31%)	.35^b^
Duration of hospital stay (days) [median (IQR)]	8 (4; 15)	9 (7; 14)	.45^a^
Transfer to the children’s hospital due to hypoglycemia	15/144 (10%)	17/95 (18%)	.12^b^
Duration of treatment with intravenous glucose due to hypoglycemia (days) [median (IQR)]	2 (2; 3)	4 (3; 5)	.02^a^
Maximum intravenous glucose (mg/kg/min) [median (IQR)]	4.6 (4.2; 5.3)	5 (3.6; 6)	.49^a^
Treatment with glucose gel	55/144 (38%)	51/95 (54%)	.02^b^

FGR, fetal growth restriction; IQR, interquartile range; SDS, standard deviation score; SGA, small for gestational age.

^a^Mann-Whitney U test, ^b^χ2 or Fisher’s Exact test; both with Bonferroni correction; ^c^statistically significant at P<.0033; ^d^statistically significant at P<.0026.

### Influence of the outside temperature on the occurrence of hypothermia in SGA and/or FGR neonates

**Figure 1:**
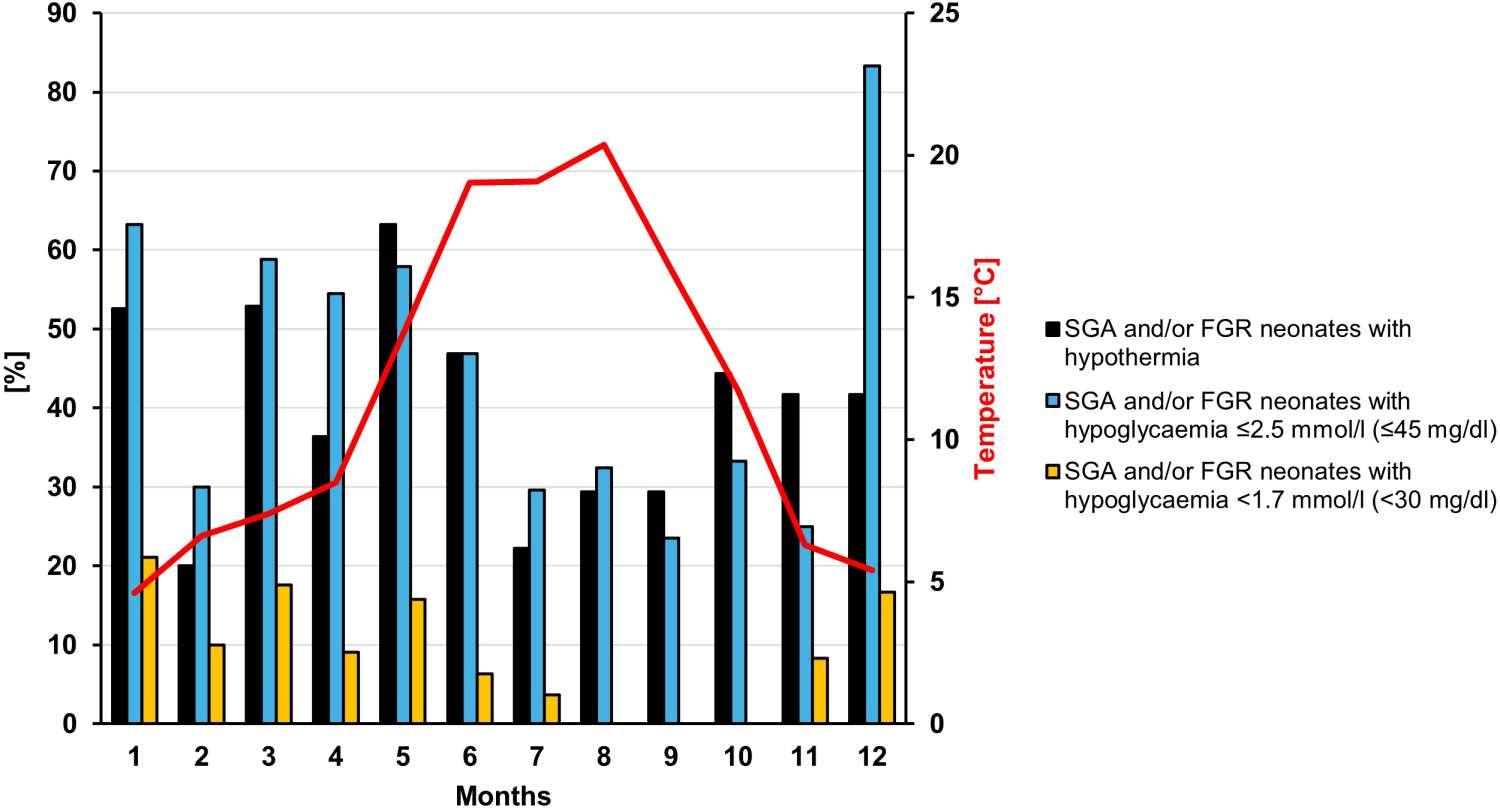
Percentage of SGA and/or FGR neonates with hypothermia and/or hypoglycemia displayed for each month. The red line shows the monthly mean outside temperature in Düsseldorf.

As the number of neonates with different risk factors varied throughout the study period, the analyses regarding the outside temperature in the birth month was only performed for the subgroup of SGA and/or FGR neonates. **Figure 1** shows the percentage of SGA and/or FGR neonates with hypothermia for each birth month and the mean monthly outside temperature. There was no direct correlation between the mean outside temperature in the birth month and the percentage of neonates with hypothermia in the corresponding month (Spearman correlation coefficient [*r*_s_] = -0.294, *P* = .35). However, the number of neonates with hypothermia was higher in the two coldest months (December/January, mean outside temperature 5.0°C) compared to the two warmest months (July/August, mean outside temperature 19.7°C) (48.4% (15/31) vs. 19.6% (16/61), P=.04).

## Discussion

In our cohort, 23.2% of all neonates had at least one hypothermic episode during the first days of life. This incidence is comparable to rates reported in studies in other high-income countries such as Canada (26.8%) and the United States (21.7%) (5, 14). Remarkably, children at-risk for hypoglycemia had a high incidence of hypothermia, despite the implementation of a routine SOP for neonatal hypoglycemia (18), which includes preventive measures with the first recommendation stating: “Keep neonate warm ≥36.5°C” and the second: “Safe skin to skin care” (18). Whereas prevention of hypothermia is one of the major goals in treatment of very preterm infants in the delivery room (20), avoidance of heat loss should not be forgotten in late preterm and term born neonates (21). Following birth, neonates are exposed to a sudden ambient temperature drop, resulting in heat loss through conduction, convection, evaporation and radiation (22). Thus, newborns are at high risk of heat loss, until sufficient endogenous temperature regulation is established (22, 23). This transition is particularly challenging in children with limited capacity for energy production, e.g. SGA or preterm neonates. Interestingly, in our study the majority of neonates were detected with hypothermia at a median age of approximately 5.5 h, by which time most neonates had already been transferred to the maternity ward or to the children’s hospital. These findings underline, that optimal thermal management should extend beyond the delivery room throughout the first days of life.

### Risk factors for hypothermia

Neonates born SGA and/or FGR, premature or with perinatal stress were at higher risk of developing hypothermia, all of which are established risk factors for this complication (5, 14, 24). On the other hand, LGA neonates had a lower risk of developing hypothermia.

No correlation was found for the average monthly outside temperature and the percentage of children who developed hypothermia. However, more children had hypothermia in the two coldest compared to the two warmest months. This is consistent with several studies especially from developing countries that have shown an association between environmental temperature and onset of neonatal hypothermia (25–27). This is supported by data showing that increasing the temperature in the operating room to 23°C compared to 20°C during a cesarean delivery can reduce the rates of hypothermia (28). Thus, it might be worth further increasing the attention to newborn temperature management during the coldest months, at least if the care units are not strictly temperature controlled/conditioned.

Interestingly, in contrast to existing literature which reports a higher incidence of hypothermia among neonates delivered via cesarean section (24), hypothermia was more frequent in neonates born vaginally in our cohort. Possible explanations could be: 1) All children who are delivered via cesarean at our facility are promptly transferred to a specific room that is constantly maintained at 30-32°C for initial care of high-risk newborns (dried off, wrapped in warm towels, and provided with a cap). In contrast, after vaginal deliveries the children are usually initially left with their mothers in the delivery room for bonding, and the provision of a hat and warm towels may be delayed. 2) Children of mothers who had a cesarean often receive more frequent care by the nursing staff in the first few days due to the immobility of the mothers: Nursing staff might be more vigilant than parents in preventing heat loss, for example by using a radiant heater for every diaper change.

### Associated consequences/problems with hypothermia

To treat hypothermia or prevent further heat loss 25.6% neonates were temporarily placed in a warming bed/incubator in the maternity ward nursery. Of note, some neonates were placed in the warming bed as a preventive measure but did not experience hypothermia. So more than one in four neonates was temporarily separated from their mother. This, in conjunction with the high rate of hypoglycemic episodes, might explain the relatively low proportion of infants in the hypothermia group who exclusively received breastmilk (3%). Conversely, reduced breastfeeding may lead to less skin-to-skin contact, which could contribute to an increased risk of hypothermia. While the direction of causality remains speculative, these findings highlight several aspects that offer room for improvement.

In our cohort, body temperature correlated positively with BG levels. Furthermore, neonates with hypothermia were at higher risk to develop hypoglycemia ≤2.5 mmol/l (≤45 mg/dl) and <1.7 mmol/l (<30 mg/dl), had more hypoglycemic episodes and lower BG levels, with a higher need for treatment with glucose gel and transfer to the children’s hospital.

To exemplarily analyze a more homogenous group in terms of hypoglycemia risk factors and other clinical characteristics, we performed a subgroup analysis on SGA and/or FGR neonates. Within this subgroup, nearly 60% of hypothermic neonates developed at least one hypoglycemic episode ≤2.5 mmol/l (≤45 mg/dl) and nearly 15% at least one hypoglycemic episode <1.7 mmol/l (<30 mg/dl) while other clinical characteristics did not differ between hypothermic and non-hypothermic SGA and/or FGR neonates.

The high rates of neonatal hypoglycemia especially in the low range of <1.7 mmol/l (<30 mg/dl) are concerning, as these have been reported to be associated with a suboptimal neurodevelopment with lower IQ scores and worse performance regarding fine motor function and visual-motor integration (7). The potential impact of concurrent hypoglycemia and hypothermia on neurodevelopmental outcomes in neonates has not yet been systematically investigated. It is even conceivable that, in the context of hypoglycemia, hypothermia may exert a neuroprotective effect, similar to its established therapeutic use in hypoxic-ischemic encephalopathy (29). However, this hypothesis remains speculative and further prospective studies are warranted to assess the interactions and synergies between hypoglycemia and hypothermia in the neonatal population. Furthermore, the direction of causality between hypothermia and hypoglycemia in neonates cannot be answered based on the available data. It is known that neonates activate non-shivering thermogenesis via brown adipose tissue and that glucose uptake in brown adipocytes is stimulated by cold exposure (30). The high glucose demand in case of hypothermia may be especially a problem in neonates with limited glycogen reserves (e.g. SGA or preterm neonates). On the other hand, in adults it was shown in a glucose clamp experiment, that hypoglycemia leads to a decrease in body temperature (31) and there are reports that an overdose of antidiabetic drugs can lead to hypoglycemia and hypothermia (32, 33). Still, in neonates it remains unclear whether hypoglycemia leads to hypothermia due to lack of energy reserves or whether hypothermia leads to hypoglycemia due to increased glucose consumption in attempt to stabilize body temperature. Both pathomechanisms are plausible and should be further evaluated in future studies, e.g. evaluating whether the implementation of combined measures to avoid heat loss during the first hours and days of life (such as increasing the cesarean operating room and delivery room temperature, safe skin-to skin care, cap, radiant heater etc.) can reduce the rate of hypothermia and associated hypoglycemia.

The fact that the possible connection between hypoglycemia and hypothermia is underrecognized in everyday clinical practice is also shown by the low rate of temperature documentation in case of hypoglycemia (35.7% at BG ≤2.5 mmol/l (≤45 mg/dl) and 53.7% at BG <1.7 mmol/l (<30 mg/dl).

## Limitation and strengths

Limitations of this study are the heterogeneity of the cohort in terms of risk factors for hypothermia and neonatal hypoglycemia. However, the large cohort allowed performing sub-group analyses for example for the SGA and/or FGR neonates with similar results to the analysis of the total study cohort regarding the association of hypothermia and hypoglycemia. Further limitation was the non-standardized temperature measurements. Whereas it is clinical routine for every neonate to undergo at least one temperature measurement in the delivery room within the first 3 hours of life, and one after transfer to the maternity ward, additional measurements were conducted at the discretion of the attending nurse. This practice resulted in considerable variability in the number of temperature assessments per neonate, suggesting that the incidence of hypothermia might have been even higher if temperature monitoring had been standardized.

Strengths of this study include the prospective design with a large cohort of 1018 neonates, including 857 neonates with different risk factors for hypoglycemia as well as a relatively large control group of 161 neonates. Furthermore, all neonates at risk were managed with a standardized hypoglycemia prevention and treatment protocol that reduces the risk of bias. Moreover, postnatal clinical course including all BG levels and interventions of each neonate were captured in high detail for statistical analysis, allowing a precise analysis of hypoglycemia in association with hypothermia.

## Conclusion

This study demonstrates that the incidence of hypothermia in late preterm and term born neonates is high, even in a high-income country and despite a rigorous hypoglycemia prevention program including recommendations for thermal management. Furthermore, hypothermia was strongly associated with more frequent and more profound hypoglycemic episodes, more frequently requiring glucose gel treatment and transfer to the children’s hospital for intravenous glucose. Expectedly, SGA and/or FGR, prematurity, and perinatal stress were risk factors for the development of hypothermia. Further prospective studies are needed to elucidate the direction of causality between hypothermia and hypoglycemia, and to assess the effectiveness of thermal management strategies in reducing both conditions. To reduce neurodevelopmental impairment upon hypoglycemia, all guidelines on neonatal hypoglycemia should include “hypothermia” as a potential risk factor or indicator for neonatal hypoglycemia, and awareness should be raised that hypoglycemia should be considered and ruled out in cases of neonatal hypothermia - and vice versa.

## Data Availability

The raw data supporting the conclusions of this article might be made available by the authors, upon reasonable request.
