# Supplementary material for: Association of neonatal hypothermia with neonatal hypoglycemia
Source: Front Endocrinol (Lausanne). 2025 Aug 27;16:1641140. doi: 10.3389/fendo.2025.1641140 (PMC12420256; doi:10.3389/fendo.2025.1641140)
Supplement: Supplementary file 1 [file Table1.docx]

| **eTable 1. Clinical characteristics of neonates with mild and moderate/severe hypothermia** | | | |
| --- | --- | --- | --- |
|  | **Mild hypothermia n=190** | **Moderate/severe hypothermia n=46** | **P** |
| Weeks of gestation [median (IQR)] | 38+4 (36+6; 39+5) | 38+3 (37+1; 39+5) | .80^a^ |
| Female | 77/190 (41%) | 21/46 (46%) | .53^b^ |
| Delivery mode |  |  |  |
| Vaginal | 89/190 (47%) | 28/46 (61%) | .09^b^ |
| Elective caesarean | 55/190 (29%) | 9/46 (20%) | .27^b^ |
| Unplanned caesarean | 46/190 (24%) | 9/46 (20%) | .57^b^ |
| Birth weight (g) [median (IQR)] | 2855 (2593; 3343) | 2975 (2465; 3341) | .82^a^ |
| Birth weight SDS [median (IQR)] | -0.78 (-1.57; 0.01) | -0.80 (-1.47; 0.09) | .68^a^ |
| APGAR 1; 5; 10 minutes [median (IQR)] | 9 (9; 9); 10 (9; 10); 10 (10; 10) | 9 (9; 9); 10 (10; 10); 10 (10; 10) | .11; .37; .07^a^ |
| Arterial cord blood pH [median (IQR)] | 7.29 (7.22; 7.32) | 7.28 (7.22; 7.32) | .45^a^ |
| Arterial cord blood base excess (mmol/l) [median (IQR)] | -4.2 (-6.6; -2.0) | -3.9 (-7.1; -1.75) | .86^a^ |
| Feeding type |  |  |  |
| Breastmilk | 6/190 (3%) | 1/46 (2%) | .99^b^ |
| Breastmilk + formula | 163/190 (86%) | 38/46 (83%) | .64^b^ |
| Formula | 21/190 (11%) | 7/46 (15%) | .45^b^ |
| At least one episode of hypoglycemia ≤2.5 mmol/l (≤45 mg/dl) | 100/190 (53%) | 26/46 (57%) | .64^b^ |
| At least one episode of hypoglycemia <1.7 mmol/l (<30 mg/dl) | 20/190 (11%) | 10/46 (22%) | .05^b^ |
| Number of BG levels between 2.6-3.0 mmol/l (46-54 mg/dl) [median (IQR)] | 1 (0; 2) | 1 (0; 2) | .64^a^ |
| Number of BG levels ≤2.5 mmol/l (≤45 mg/dl) [median (IQR)] | 1 (0; 1) | 1 (0; 1) | .77^a^ |
| Lowest measured blood glucose (mmol/l) [median (IQR)] [mg/dl] | 2.4 (2.1; 2.9) [44 (37; 53)] | 2.5 (1.9; 2.8) [45 (34; 51)] | .57^a^ |
| Treatment at the children’s hospital | 59/190 (31%) | 17/46 (37%) | .48^b^ |
| Duration of hospital stay (days) [median (IQR)] | 7 (4; 11) | 6 (4; 12) | .82^a^ |
| Transfer to the children’s hospital due to hypoglycemia | 30/190 (16%) | 11/46 (24%) | .20^b^ |
| Duration of treatment with intravenous glucose due to hypoglycemia (days) [median (IQR] | 3 (2; 4) | 4 (3; 6) | .34^a^ |
| Maximum intravenous glucose dosage (mg/kg/min) [median (IQR)] | 4.7 (4.2; 5.4) | 5.1 (3.0; 5.9) | .64^a^ |
| Treatment with glucose gel | 98/190 (52%) | 24/46 (52%) | .94^b^ |
| Abbreviations: IQR, interquartile range; SDS, standard deviation score  ^a^Mann-Whitney U test, ^b^χ2 or Fisher's Exact test; both with Bonferroni correction | | | |

| **eTable 2. Clinical characteristics of controls and neonates with hypothermia as the only risk factor** | | | |
| --- | --- | --- | --- |
|  | **Control neonates**  **without hypothermia n=161** | **Control neonates**  **with hypothermia n=28** | **P** |
| Weeks of gestation [median (IQR)] | 39+1 (38+4; 40+1) | 38+5 (37+6; 39+6) | .13^a^ |
| Female | 82/161 (51%) | 14/28 (50%) | .99^b^ |
| Delivery mode |  |  |  |
| Vaginal | 50/161 (31%) | 9/28 (32%) | .99^b^ |
| Elective caesarean | 90/161 (56%) | 13/28 (46%) | .41^b^ |
| Unplanned caesarean | 21/161 (13%) | 6/28 (21%) | .09^b^ |
| Birth weight (g) [median (IQR)] | 3460 (3193; 3805) | 3300 (3083; 3521) | .03^a^ |
| Birth weight SDS [median (IQR)] | 0.04 (-0.39; 0.64) | -0.18 (-0.71; 0.26) | .06^a^ |
| APGAR 1; 5; 10 minutes [median (IQR)] | 9 (9; 9); 10 (10; 10); 10 (10; 10) | 9 (9; 9); 10 (10; 10); 10 (10; 10) | .57; .66; .011^a^ |
| Arterial cord blood pH [median (IQR)] | 7.30 (7.25; 7.33) | 7.29 (7.24; 7.33) | .45^a^ |
| Arterial cord blood base excess (mmol/l) [median (IQR)] | -2.4 (-5.3; -1.4) | -3.6 (-6.2; -1.4) | .41^a^ |
| Feeding type |  |  |  |
| Breastmilk | 37/161 (23%) | 0/28 (0%) | .002^b,c^ |
| Breastmilk + formula | 108/161 (67%) | 27/28 (96%) | <.001^b,c^ |
| Formula | 16/161 (10%) | 1/28 (3%) | .48^b^ |
| At least one episode of hypoglycemia ≤2.5 mmol/l (≤45 mg/dl) | 39/161 (24%) | 11/28 (39%) | .11^b^ |
| At least one episode of hypoglycemia <1.7 mmol/l (<30 mg/dl) | 2/161 (1%) | 1/28 (4%) | .38^b^ |
| Number of BG levels between 2.6-3.0 mmol/l (46-54 mg/dl) [median (IQR)] | 0 (0; 1) | 2 (0; 2) | <.001^a,c^ |
| Number of BG levels ≤2.5 mmol/l (≤45 mg/dl) [median (IQR)] | 0 (0; 0) | 0 (0; 1) | .04^a^ |
| Lowest measured blood glucose (mmol/l) [median (IQR)] [mg/dl] | 3.1 (2.6; 3.6) [56 (47; 64)] | 2.6 (2.2; 2.9) [46 (40; 52)] | <.001^a,c^ |
| Treatment at the children’s hospital | 3/161 (2%) | 5/28 (18%) | .002^b,c^ |
| Duration of hospital stay (days) [range] | 3-4 | 3-7 | .23^a^ |
| Transfer to the children’s hospital due to hypoglycemia | 3/161 (2%) | 3/28 (11%) | .04^b^ |
| Treatment with glucose gel | 29/161 (18%) | 12/28 (43%) | .006^b^ |
| Abbreviations: IQR, interquartile range; SDS, standard deviation score  ^a^Mann-Whitney U test, ^b^χ2 or Fisher's Exact test; both with Bonferroni correction; ^c^statistically significant at P<.0042 | | | |
